# A randomised controlled feasibility study of interpersonal art psychotherapy for the treatment of aggression in people with intellectual disabilities in secure care

**DOI:** 10.1186/s40814-020-00703-0

**Published:** 2020-11-19

**Authors:** Simon S. Hackett, Ania Zubala, Katie Aafjes-van Doorm, Thomas Chadwick, Toni Leigh Harrison, Jane Bourne, Mark Freeston, Andrew Jahoda, John L. Taylor, Cono Ariti, Rachel McNamara, Lindsay Pennington, Elaine McColl, Eileen Kaner

**Affiliations:** 1 Newcastle University, Faculty of Medical Sciences, Newcastle upon Tyne, UK; 2 Cumbria, Northumberland, Tyne & Wear NHS Foundation Trust, Newcastle upon Tyne, UK; 3 University of the Highlands and Islands, Inverness, UK; 4 Yeshiva University, New York, USA; 5 Glasgow University, Glasgow, UK; 6 Northumbria University, Newcastle upon Tyne, UK; 7 Centre for Trials Research, Cardiff University, Cardiff, UK

## Abstract

**Background:**

Rates of aggression in inpatient secure care are higher than in other psychiatric inpatient settings. People with intellectual disabilities in secure care require adapted psychological treatments. Interpersonal art psychotherapy incorporates the use of creative art making approaches by participants, thus reducing sole reliance upon verbal interactions during psychotherapy for people who may have communication difficulties. During interpersonal art psychotherapy, participants are individually supported by their therapist to consider how they conduct relationships. This includes the influence and impact of interpersonal issues resulting in repeated patterns of conflict. The key feasibility objectives were to assess recruitment and retention rates, follow-up rates and trial procedures such as randomisation, allocation and identifying any practical or ethical problems. In addition, a preliminary ‘signal’ for the intervention was considered and an indicative sample size calculation completed. The acceptability of a potential third trial arm attentional control condition, mindful colouring-in, was assessed using four single-case design studies and a UK trial capacity survey was conducted.

**Methods:**

Adult patients with intellectual disabilities in secure care were recruited and randomised to either interpersonal art psychotherapy or delayed treatment in this multi-site study. Outcomes were assessed using weekly observations via the Modified Overt Aggression Scale and a range of self-report measures. Within study reporting processes, qualitative interviews and a survey were completed to inform trial feasibility.

**Results:**

Recruitment procedures were successful. The target of recruiting 20 participants to the trial from multiple sites was achieved within 8 months of the study opening. All participants recruited to the treatment arm completed interpersonal art psychotherapy. Between-group differences of interpersonal art psychotherapy versus the delayed treatment control showed a ‘signal’ effect-size of .65 for total scores and .93 in the verbal aggression sub-scale. There were no amendments to the published protocol. The assessment of key feasibility objectives were met and the trial procedures were acceptable to all involved in the research.

**Conclusion:**

This study suggested that a randomised controlled trial of interpersonal art psychotherapy is acceptable and feasible.

**Trial registration:**

ISRCTN14326119 (Retrospectively Registered).

## Key messages

1. What uncertainties existed regarding the feasibility?
  Recruitment, retention and follow-up rates.
  Trial procedures.
  Indicative sample size and capacity for a RCT in secure care.
2. What are the key feasibility findings?
  Recruitment, retention and follow-up rates were acceptable.
  Trial procedures were viable with requirements for minor adjustment only.
  A randomised controlled trial of interpersonal art psychotherapy is acceptable and feasible.
3. What are the implications of the feasibility findings for the design of the main study?
  The reduction of aggression is a primary outcome.
  An attentional condition could be included as a potential third arm in a future trial.
  Within-trial cost-effectiveness analysis can be conducted.

## Background

Within England, estimates suggest 3035 (0.3%) of people who have an intellectual disability are receiving treatment in psychiatric hospital settings’ with half being treated in inpatient secure care settings’ [1]. The presence of a co-occurring mental illness can significantly increase the likelihood of people with intellectual disabilities having experienced victimisation and/or committing offences [2]. Secure care, including within the NHS, provides treatment for adults with mental illness, personality disorder and neurodevelopmental disorders including intellectual disability and autism [3]. Patients with intellectual disability being treated in secure care wards are more likely to have a long stay in hospital, defined as more than 10 years in high secure, 5 years in medium secure or 15 years in a mix of high and medium secure settings, compared to patients in other types of secure mental health wards [4]. The health expenditure for adults with intellectual disability in secure care is estimated to be over 300 million pounds sterling per annum [1].

A systematic meta-analysis of violence in psychiatric settings (23,972 patients) reported that the proportion of patients who committed at least one act of violence was 17% (95% confidence interval (CI) 14–20%) [5]. Factors associated with higher rates of violence and aggression in inpatient psychiatric settings include there being a higher proportion of male patients; involuntary patients; patients with a diagnosis of schizophrenia; and patients with alcohol use disorder [5]. In community residential settings, factors associated with aggression are similar and include lifetime substance and alcohol misuse, a history of violence and patients having a diagnosis of a personality disorder [6]. Previous studies have reported patients in secure care/forensic as being more likely to be violent than those in other types of psychiatric units [7]. People with intellectual disability who are inpatients in services and/or residential care are more likely to have reported aggression compared with people living independently [8]. Studies of individuals with moderate intellectual disability who live in a residential facility have shown that in most cases, aggressive behaviour was positively or negatively reinforced by social and task-related events [9, 10]. Interpersonal perceptions and dynamic relational factors have been identified as having a role in determining staff responses [11]. Greater sensitivity in interpersonal situations has been identified as contributing to the likelihood of aggression in some people with intellectual disabilities [12, 13]. Problems of aggression might partly be exacerbated by a tendency towards perceiving hostility in others, heightened emotional arousal and personal experiences of conflict [14–16].

A systematic review (2019) of randomised controlled trials (RCT) of psychological interventions offered to forensic/secure mental health inpatients (*n* = 9 studies including 523 participants) reported that current practice is based on limited evidence with no consistent significant findings [17]. Study sample sizes ranged from 14 to 112. A low risk of bias assessment indicated that good quality RCTs can be undertaken within inpatient medium to high secure forensic settings. Economic evaluations were not included in the studies. The review concluded that further studies are needed to clarify the evidence base [17].

The most recent summary of research with offenders who have intellectual disability (2018) identified the development of effective interventions for this vulnerable group as a priority [18]. Adaptations are required for psychological interventions for people who have intellectual disabilities and mental health problems [19] with specific recommendations for adult patients in secure care [3].

National practice-based guidelines have been developed for art therapy with people who have an intellectual disability [20] as an approach that places less of a burden on verbal communication within psychotherapy. A systematic review of the clinical effectiveness of art therapy amongst people with non-psychotic mental health disorders [21] identified 15 RCTs (*n* = 777), but excluded people with intellectual disabilities. Improvements to the design of future art therapy trials were considered to be the inclusion of non-active treatment as usual/wait-list control arm, attentional (art and craft activity) control and/or an active psychological comparator [21]. The report concluded that art therapy showed positive effects and an indication of cost-effectiveness compared to wait-list control [21]. Interpersonal art psychotherapy has been developed for use with adults who have mild/moderate intellectual disability in secure care [22–26].

### Aims

1. To test the feasibility of conducting an RCT evaluating interpersonal art psychotherapy in NHS secure care, identifying:
  Recruitment and retention rates
  Follow-up rates
  Trial procedures (randomisation and allocation to delayed treatment waiting-list, measurement and fidelity) generating any practical or ethical problems
2. To obtain a preliminary ‘signal’ for the intervention and indicative sample size calculation.
3. To assess the acceptability of a potential third trial arm attentional control condition, mindful colouring-in.
4. To survey NHS secure care sites to assess capacity for a future definitive trial.

## Methods

All procedures in this study received NHS ethical approval from the Health Research Authority (IRAS project ID: 191223, REC reference: 16/NE/0220). The full study protocol was published in an open access journal in October 2017 [26]. Informed consent was obtained from patients before assessments and/or intervention procedures were conducted.

## Study design

### RCT

This was a multi-site, 1:1 randomised controlled feasibility study, with participants being randomised to either interpersonal art psychotherapy plus usual care or usual care with delayed treatment (waiting-list).

### Single-case design

To inform future trial design and the potential for inclusion of an attentional control condition [27–29], four separate single-case design (SCD) studies were run in parallel to the RCT. Mindful colouring-in was chosen and piloted as a novel art-based attentional condition [30] followed by an assessment of the acceptability of the intervention [31, 32]. An ‘ABACA’ design was used [33] *A*(1) = baseline; *B* = mindful colouring-in; *A*(2) = monitoring; *C* = interpersonal art psychotherapy; *A*(3) = post-therapy. One-to-one semi-structured qualitative interviews were completed with participants and the findings from thematic analysis [34] were mapped against a treatment acceptability framework [32].

### Survey

A survey of NHS secure care providers in the UK was carried out to ascertain capacity and interest in participation in a future trial.

## Feasibility objectives

The feasibility objectives included:

1. Recruitment, such as patients’ willingness to be randomised and clinicians’ willingness to recruit their patients into the study; assessed by the recruitment target being met for the number of consented participants during enrolment (*n* = 20).
2. Identifying ethical issues related to seeking informed consent and risks of coercion, including the potential for patients to participate in the study believing it will positively or negatively influence their inpatient treatment or detention under the Mental Health Act [35]; assessed during enrolment from the number of patients agreeing and/or declining to participate and the responses collected from patients declining to participate after receiving study participant information.
3. Suitability of procedures and materials, including study information, outcome measures, burden of outcome measures/validated tools, data collection and maintenance of data integrity from multiple study sites; assessed by local research assistants reporting feedback from participants at each data collection point (baseline, post-, follow-up) on the burden of outcome questionnaires. The level of completion of questionnaires (instrument and item response rates) was monitored and recorded within routine data integrity checks.
4. Describing routine care/treatment as usual, identifying characteristics of treatment as usual and individualised patient care pathways across multiple sites, within high and medium/low security; assessed via an inventory of participant care plans found within medical records. Checks were conducted by research assistants at baseline for all enrolled participants across multiple-sites.
5. Attrition and acceptability, including rates of attendance for treatment, reasons for non-attendance and/or drop-out and lack of retention for data collection at the follow-up points; assessed by study therapists recording participant attendance/non-attendance to treatment sessions, research assistants recording participant reasons for drop-out (including those in the delayed treatment wait-list arm), and rates of retention for follow-up data collection.
6. Identifying risks of contamination, such as participants on the waiting-list/delayed treatment arm receiving active components of the treatment during routine care; assessed at baseline by completion of an inventory of participant care plans carried out by research assistants for all enrolled across multiple-sites. An ongoing review of changes in  care provision for participants in the treatment arm was completed by study therapists within 18 weeks (concurrent with interpersonal art psychotherapy).
7. Treatment fidelity, identifying therapist adherence with the required activity in the treatment manual and piloting treatment fidelity measure; assessed by measures of inter-rater reliability applied within treatment fidelity checks, random selection and treatment fidelity checklist assessment of audio recordings of treatment sessions (*n* = 27 session recordings), and post-study qualitative group interviews with all study therapists to capture their responses to delivering the manualised intervention.
8. Acceptability of a novel attentional condition; assessed in four separate single-case design studies with data collected via post-treatment participant interviews.

## Participants

### RCT (feasibility)

Patients took part in the RCT from three secure hospitals in England (Nottinghamshire, Lancashire, and East London) from February 2017 with all data collection completed by February 2019. The study aimed to recruit 10 participants to each arm (*n* = 20) with the study being focused upon assessing feasibility across multiple-sites. Participant recruitment targets were met within 8 months of the study opening.

### Eligibility

Secure care patients were eligible for inclusion if they were an adult the age of 18 and over, an inpatient in a NHS secure hospital, with an IQ of between 55 and 79 (within a range including moderate to mild intellectual disability and borderline intellectual functioning), and able to give informed consent [36]. The patient’s involvement in the study required support from their responsible clinician and/or multi-disciplinary team (MDT). Patients were considered eligible if they had a clinical profile that included a historic and/or continuing presentation of emotional control difficulties and/or observed aggression.

Patients were excluded if they were deemed unable to give informed consent, had no clinical indicators for the psychotherapeutic treatment in their clinical profile, had a planned discharge from hospital within 12 months of the start of the study or were undergoing medication dose titration for the treatment of acute psychotic symptoms. The inclusion and exclusion criteria remained unchanged throughout the study. Patient eligibility checking was carried out by study therapists followed by detailed screening based upon all of the study inclusion and exclusion criteria.

## Interventions

### Interpersonal art psychotherapy

Interpersonal art psychotherapy has been developed as a brief structured manualised psychological therapy for people with mild to moderate intellectual disabilities [22, 23, 25, 26]. It incorporates creative art approaches to enhance engagement and aid understanding [20]. The interpersonal component of the treatment manual is informed by the core conflictual relationship theme (CCRT) approach, the CCRT being the central relationship pattern, script, or schema that each person might follow in conducting their relationships [23, 25]. The therapy includes exploration of themes associated with the participants’ own accounts of their interactions with people [37].

Interpersonal art psychotherapy consisted of 15 individual weekly 1-h sessions provided by a UK Health and Care Professions Council (HCPC) art therapist with experience of working in secure care with people who have intellectual disabilities. A therapist worked with one patient for the duration of treatment with a target of completing all of the therapy topics within an 18-week period. The therapy topic session schedule is as follows, sessions 1 to 3 personal goals, coping responses and self-management; 4 to 5 relationships; 6 to 10 life events; 11 to 12 interpersonal themes; 13 to 15 imagined future and final review. The structure of each therapy session is as follows; the therapist introduces the session content, agenda setting, a directed art activity (as determined by the manual and session schedule) and a reflective discussion about the art activity. Therapists ‘augment’ reflective discussions by using supplied resources or creating additional visual material to aid communication and understanding.

Study therapists completed 2-days of training which included familiarization with the manual, formal teaching, group discussion and rehearsal/role-play. During the study, individual clinical supervision was provided on a fortnightly to monthly basis. For the purpose of completing treatment fidelity checks, audio recordings of therapy sessions were reviewed by the research team against a treatment fidelity measure. The interpersonal art psychotherapy checklist was developed and piloted separately by two raters resulting in agreement on four criterion recordings (SH and AZ). Inter-rater discussion and consensus rating led to further refinement of checklist for inter-rater training purposes. Prior to full assessment of treatment fidelity within the study, the checklist (34 items in total, 29 items scoring on a three point categorical ordinal scale) was assessed against a sample of recordings selected from different therapists, different clients and different time points (2 early, 1 middle, 1 late therapy). Inter-rater reliability testing was conducted by two independent raters (AZ and TLH) with agreement ranging from 72.1% at initial unguided rating to 88.3% at second rating following inter-rater reflective training. Both Kappa and weighted Kappa were calculated [38, 39] with Kappa below 0.60 taken to indicate inadequate agreement [40]. Kappa values were calculated from all independently rated recordings at two stages of inter-rater reliability testing. At initial unguided rating, mean Kappa was 0.52 and weighted Kappa was 0.65, whilst at rating following training, the mean values were 0.80 and 0.85 respectively.

### Usual care (delayed treatment)

Usual care within inpatient secure settings involves assessment and treatment by a specialist MDT using the Care Programme Approach (CPA) [3] to coordinate and plan care. MDTs comprise psychiatrists, clinical and forensic psychologists, mental health and intellectual disability nursing staff and allied health professionals (AHPs), for example, occupational therapists, arts therapists and speech and language therapists. The work of MDTs includes risk assessment/formulation and management, recovery focused care and/or positive behaviour support (PBS) [3]. Access to psychotherapy/psycho-educational work includes anger management and anger maintenance, emotions group, drug and alcohol work, speech and language therapy and/or communication group, art therapy group, relaxation, sex education and specific offence related treatment, such as sex offender treatment. Pharmacotherapy treatment and review include (where required) the prescription of mood stabilisers, antipsychotic medication, stimulant medication (for the treatment of attention deficit hyperactivity disorder) and rapid tranquilising medication, pro re nata (PRN).

An inventory of participants’ care plans (treatment as usual) was completed. Assessment of care-coordination, MDT members and the provision of bio-psycho-social components of treatment [3] were shown to be present and provided to participants across study sites. Two participants in the delayed treatment waiting-list continued to attend a weekly art therapy group. No participants in the intervention arm received additional group art therapy.

### Randomisation and allocation

**Fig. 1:**
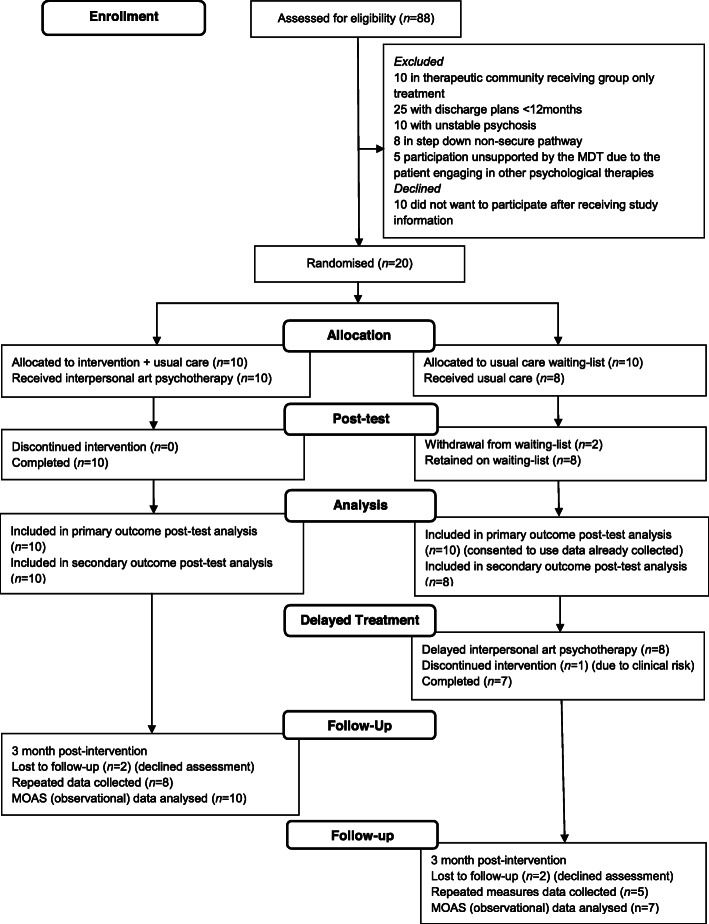
CONSORT flow diagram

Consenting patients were individually randomised and assigned across both allocations. Statistical support was provided within the study (TC). Simple randomisation was used to generate the allocation sequence using a computer statistical package (IBM SPSS Statistics version 25 ©). The concealed sequence was retained by a research assistant who was independent from the recruitment process at each study site. As blinding to treatment was not possible in this study, allocation concealment was maintained for participants following baseline assessment. Local study therapists and research staff conducting enrolment were blinded at baseline assessment and then requested the allocation via email contact with the research assistant on a participant by participant basis. Participants were then informed if they had been assigned to either interpersonal art psychotherapy or to usual care delayed treatment. A CONSORT diagram detailing recruitment, allocation, delayed-treatment and retention is shown in Fig. 1.

## Measures

The Modified Overt Aggression Scale (MOAS) [41] is an observer-rated treatment outcome measure for people with intellectual disabilities. The MOAS measures both the frequency and severity of aggressive behaviour (< 10 or > 10 observations) in the previous 7 days. It measures four types of aggression, namely (a) verbal aggression, (b) physical aggression against objects, (c) physical aggression against self and (d) physical aggression against other people. Agreement between raters for MOAS total scores is high (intra-class correlation coefficient (ICC)) of 0.93, verbal aggression (ICC = 0.90) and physical aggression against others (ICC = 0.90) [41]. The MOAS was collected on a weekly basis by research assistants for the duration of the study, providing extended post-treatment follow-up data.

The following self-report measures were administered by research assistants (being blinded to allocation) at baseline. Assessments were then repeated at post-test (between 15 and 18 weeks at end of treatment or prior to the start of delayed treatment) and at 12 weeks post-treatment follow-up. Research assistants were asked to report upon participants’ responses to these measures, for example, the overall burden of assessment and when participants required additional prompting or explanations for item questions.

The Brief Symptom Inventory (BSI) [42] is a 53-item self-report inventory with good internal consistency (Cronbach’s alpha: 0.84), sensitivity of 82%, specificity of 75% and provides information regarding symptom distress on a range of psychological symptoms. Sub items can be calculated for a Global Severity Index (GSI), Positive Symptom Total (PST) and Positive Symptom Distress Index (PSDI). The BSI was administered to both arms by research assistants using an ‘assisted completion format’ [43, 44].

The Novaco Anger Scale (NAS) [45] is a 48-item self-report questionnaire designed to index a person’s disposition for anger (a risk factor for aggression). The NAS has internal reliability (alpha) of .95 and a 2-week test-retest reliability of .84. Subscales are included for ‘cognitive’ composed of items operationalising justification, rumination, hostile attitude and suspicion; ‘arousal’—intensity, duration, somatic tension and irritability; ‘behavioural’—impulsive reaction, verbal aggression, physical confrontation and indirect expression.

The Glasgow Anxiety Scale for people with intellectual disability (GAS-ID) [46] is a 27-item self-rating scale of anxiety-symptoms for people who have a mild intellectual disability. The maximum possible score on this scale is 54, with subtotals for component scales. Internal consistency for the GAS-ID total scores is reported to be 0.96, and subscales ‘worries’ 0.92, ‘fears’ 0.80 and ‘physiological symptoms’ 0.90. Test-retest reliability is reported to be 0.95 [46].

ICEpop CAPability Quality of Life measure for Adults (ICECAP-A V2) [47, 48] assesses capability (what an individual can do) rather than functioning (what they actually do) to avoid imposing a particular idea of what a good life constitutes and to reflect the importance of freedom to choose [49]. Components include attachment (an ability to have love, friendship and support), stability (an ability to feel settled and secure), achievement (an ability to achieve and progress in life), enjoyment (an ability to experience enjoyment and pleasure) and autonomy (an ability to be independent). This conceptualisation of wellbeing may fit more appropriately with accurate assessment of the personal circumstances of patients detained in secure care. The ICECAP-A was included within this feasibility study to assess if it could be used within an intellectual disability population and included in cost-effectiveness analysis in a future trial. The ICECAP-A cannot be used to elicit quality adjusted life tears (QALYs) but has been suggested for the purpose of economic evaluation focusing on full or sufficient capability [50, 51].

Therapy process observations were completed by study therapists at the first therapy session and at 3-week intervals following the start of treatment using the Working Alliance Inventory (WAI-Therapist) [52]. The WAI is one of the most frequently used instruments in the therapeutic alliance literature [53] consisting of three subscales: affective bond, agreement on tasks and agreement on goals. The subscales provide separate scores for each of the three domains they measure and a global score of the working alliance. Items are rated on a Likert scale ranging from 1 (never) to 7 (always) with 2 (rarely), 3 (occasionally), 4 (sometimes), 5 (often) and 6 (very often) between the two extremes. Various studies of the Working Alliance Inventory-Observer scale (WAI-O) [54] have demonstrated high internal consistency (α = .93) [55], predictive validity and interrater reliability, e.g. an intraclass correlation coefficient of .92 in [54].

## Enrolment

Study information was shared with clinical staff and MDTs within the secure care settings including letters to responsible clinicians providing study information and the inclusion and exclusion criteria. Patients were provided with study information and if they wished to participate, they were invited to have a member of staff ‘who knows them well’ present as a witness. Where needed, patients were read the study information and consent form [56]. Additional checks [36] were completed if any concerns were raised regarding a patient’s capacity to consent by either the patient, their responsible clinician and/or a member of the MDT or a member of the local research team. Patients were given a minimum of 48 h to decide if they would like to participate after receiving study information. Baseline measures were administered to consenting patients prior to randomisation allocations being shared.

To assess the feasibility of trial procedures, an email reporting system to the chief investigator was in place alongside routine trial meetings with members of the study team. Routine supervision of therapists delivering the manualised treatment took place throughout the study. Individual debriefing sessions with research assistants took place and post-study group interviews were conducted with all study therapists.

## Data analysis

Summary statistics are presented for each outcome measure separately by arm at each study time point (baseline and post-test). Summary statistics for the difference between post-test and baseline time points are also presented within each trial arm. Magnitudes of differences are reported using Cohen’s *d*, along with corresponding 95% CI [57]. ANOVA was used to explore small data-sets, for example, the Working Alliance Inventory (WAI). Measures of inter-rater reliability were applied within treatment fidelity checks. Qualitative thematic analysis of interview data [34] was used for study therapists responses to the delivering the manualised intervention and to assess SCD participants perceptions of acceptability of an attentional condition [32]. In addition, descriptive statistics were applied to survey data.

## Results

### RCT demographic information

Table 1 shows baseline demographic information between groups. All participants were being treated in secure care, ranging from high (20%), medium (55%) and low (25%) secure hospitals with average length of stay being 5 years and 9 months, ranging from 11 months to 18 years. The largest ethnic groups in the study were White British (60%) and Black British (25%). Most participants had a mild intellectual disability (85%).

**Table 1:** Baseline demographic comparison between groups and single-cases

		IAP (*n* = 10) Mean (SD) or count	Waitlist (*n* = 10) Mean (SD) or count	Single-case Design (*n* = 4) Years, months, or count
Gender	Men	8	10	4
	Women	2	–	–
Age (years)		33.2 (10)	31.4 (7.5)	20,22,23,23
Secure care	High	2	2	–
	Medium	5	6	3
	Low	3	2	1
Time in hospital (months)		63 (46)	79 (72)	5,9,33,34
Ethnicity	White British	6	6	3
	White Irish	–	–	1
	Black British	4	1	–
	British Indian	–	1	–
	Black African	–	1	–
	Black Caribbean	–	1	–
Intellectual disability	Borderline intellectual functioning	–	–	–
	Mild	8	9	2
	Moderate	2	1	–
Additional diagnosis	Schizophrenia/paranoid schizophrenia	1	5	–
	Antisocial personality disorder	1	–	–
	Alcohol dependence syndrome	1	–	–
	Autistic spectrum disorder	2	–	1
	Attention-deficit hyperactivity/kinetic disorder	1	–	1
	Head injury	–	1	–
	Sensory neural deafness	–	1	–
	None	4	3	2
Criminal/index offence	Sexual offence/assault	4	2	2
	Physical assault/affray	3	3	1
	Criminal damage	1	1	–
	Arson	1	2	–
	Theft/burglary	–	2	1
	Murder	1	–	–

*IAP* interpersonal art psychotherapy

### RCT feasibility objectives

The target for recruitment of 20 participants across three medium-high secure hospitals was achieved (see Fig. 1 CONSORT diagram). Members of the clinical teams across multiple-sites were willing to support recruitment to the study with the exception of patients who were already actively engaging in psychological therapies in their clinical treatment (*n* = 5). Similarly, patients included in a therapeutic community programme in one secure hospital were not permitted to take part in individual psychological therapies in addition to their group based treatment programme (*n* = 10). A total of ten patients declined to participate with some stating that they did not want to interrupt their current timetable of activities. Study information was suitable for the patient group. Procedures for gaining consent were carried out with no changes to the published protocol [26].

All participants allocated to interpersonal art psychotherapy completed their treatment (*n* = 10). Combined completion rates for participants who started interpersonal art psychotherapy (including those having delayed treatment) was 94% (17/18) with one participant in high secure hospital being withdrawn from treatment due to having restricted access to pens and pencils. At post-test, participant retention across both arms was 90% (18/20). High completion rates for the MOAS [41] observational measure led to 100% (20/20) of the target primary outcome data being collected and analysed at post-test and 85% (17/20) at follow-up (for both arms). Participants on the waiting-list had a 20% (2/10) drop-out rate prior to starting their delayed treatment. Participant drop-out from the delayed treatment group was reported as being primarily due to deteriorating mental health. Participants allocated to fifteen sessions of interpersonal art psychotherapy were required to remain in the study for approximately 8 months, with a 20% (2/10) loss to follow-up for secondary outcome repeated measures. Participants who were allocated to the usual care, received delayed interpersonal art psychotherapy and assessed at follow-up 3-month post-treatment were required to remain in the study for up to 12 months.

Maintaining full data-collection integrity across multiple-sites was not fully achieved due to procedures not being carried out correctly at one site (secondary outcome measures were not collected at baseline from two participants in the delayed treatment arm). Due to limited resources in this study, capacity for live data-collection monitoring was not possible. Four participants declined to complete secondary outcome measures at follow-up assessment.

Observations of burden of assessment were reported by research assistants. Administration of the BSI was conducted using an assisted completion format [43] with some participants needing more than one meeting with the research assistant to complete all items. Participants were found to have no difficulty with completion of the GAS-ID and NAS. Research assistants administering the tests reported that some participants struggled to understand some items of the ICECAP-A assessment, which were abstract in concept and required further explanation.

### Treatment fidelity

Assessment procedures for treatment fidelity and manual adherence were completed using the interpersonal art psychotherapy checklist, with therapists having been instructed to audio record all treatment sessions during the study. The treatment fidelity assessment was conducted from 27 randomly selected recordings (8 early, 11 middle, 8 late therapy). Study therapists were assessed as having 82% adherence to the interpersonal art psychotherapy manual.

### Study therapist group interviews

Two post-study group interviews were carried out with all study therapists. Interview schedules focused on their experiences of intervention delivery [58] and questions were informed by an implementation science approach [59]. Qualitative thematic analysis [34] of transcriptions from the group discussion were completed.

The therapists’ responses to treatment fidelity procedures and manual adherence checks indicated that therapists gained confidence during the study in following the treatment manual instructions. Therapists reported that the recording of therapy sessions for treatment fidelity checks within a secure hospital context raised concerns for some participants, who associated audio-recording devices with the experience of police interviews and required additional reassurance.

### Preliminary ‘signal’ of the efficacy of the intervention

Effect-size estimates (Cohen’s *d*) for baseline-post-test difference between groups are shown in Table 2. Between group differences at post-test comparison of the interpersonal art psychotherapy versus the delayed treatment control showed an effect-size of .65 (− 0.25–1.54, 95% CI) for total scores and .93 (0.01–1.85, 95%CI) in the verbal aggression sub-scale. The Brief Symptom Inventory–Positive Symptom Distress Index (BSI-PSDI) showed a between group effect-size of .79 (− 0.20–1.79, 95% CI).

**Table 2:**
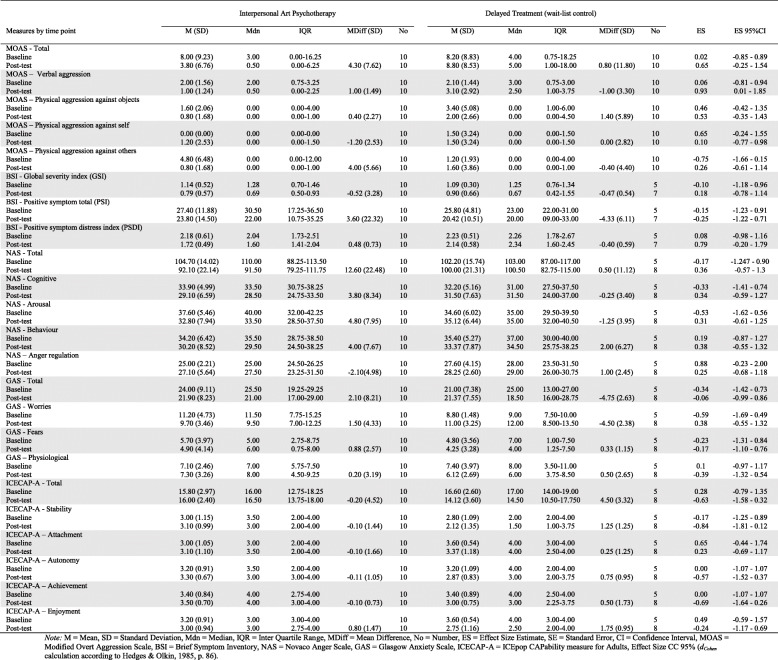
Descriptive statistics for baseline and post-test difference and effect size estimates

*M* mean, *SD* standard deviation, *Mdn* median, *IQR* interquartile range, *MDiff* mean difference, *No* number, *ES* effect size estimate, *SE* standard error, *CI* confidence interval, *MOAS* Modified Overt Aggression Scale, *BSI* Brief Symptom Inventory, *NAS* Novaco Anger Scale, *GAS* Glasgow Anxiety Scale, *ICECAP*-*A* ICEpop CAPability measure for Adults, Effect Size CC 95% (*d*_Cohen_ calculation according to Hedges & Olkin, 1985, p. 86)

**Fig. 2:**
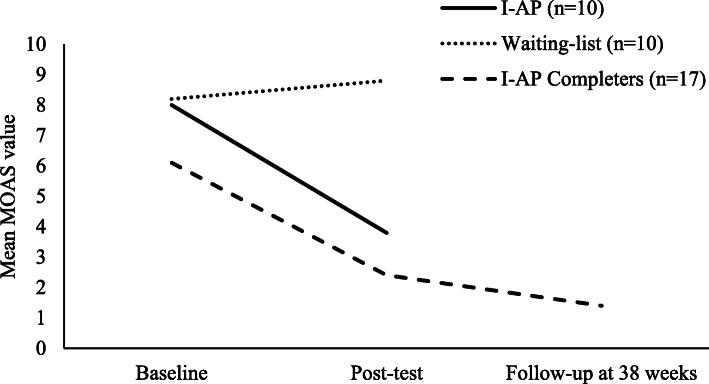
Mean MOAS scores for groups and interpersonal art psychotherapy completers (combined data).

Pooled MOAS data from all interpersonal art psychotherapy completers (*n* = 17) (combined scores from both arms including delayed treatment participants) can be seen in Fig. 2. Mean MOAS scores showed a reduction from baseline *M* = 6.1 (SD = 8–1) to post-test *M* = 2.4 (SD = 5.4), and follow-up *M* = 1.4 (SD = 3.5).

For seven patients, the Working Alliance Inventory (WAI) ratings were completed for five of the timepoints; for the other 11 patients, the data were incomplete. Based on the available data, the repeated measure ANOVA indicated a positive change in working alliance over treatment, *F* = 2.976 (4), *p* = .036. Overall, within this small sample, alliance was not significantly related to any of the outcome measurements. Participants with more severe psychopathology (as measured on the BSI-GSI) had a lower working alliance throughout the treatment than the other participants.

### Indicative sample size calculation

For the MOAS primary outcome measure, we conducted a sample size calculation assuming power of 90% and a type I error rate of 5%. Based on the results of the feasibility study, we also assumed it would be important to detect a clinically important difference of 5 points on the MOAS scale and we assumed a common SD in the control and intervention groups of 10 points. In addition, to account for the fact that a single therapist would apply the intervention to more than one participant, we made the following assumptions to account for this type of clustering. It was assumed that in a future trial, each site would have a minimum of 2 therapists and the intraclass correlation (ICC) of the MOAS scores for the same therapist would be 1%. This is consistent with assumptions used in similar studies. Using these assumptions as a basis, we calculated that 96 participants would be required in each group. Allowing for an assumed attrition rate of 20%, the study would require 120 participants in each group or a total of 240 participants.

This would also allow the study to detect a difference of 0.5 points on the change in BSI-positive symptom distress index to be detected as a secondary outcome, using a SD estimate of 0.8. Based on the sample size calculated above, this would result in a power in excess of 90% for the BSI comparison.

### Attentional condition acceptability (single-case design)

For the assessment of the acceptability of an attentional control condition (art and craft activity/active psychological comparator), participants (*n* = 4) were offered up to 15 sessions (up to 1 h) of mindful colouring-in [30, 60, 61] facilitated by a qualified art psychotherapist. A convenience sample (non-randomised) of four patients from a secure NHS hospital (Northumberland) with medium-low secure wards, see demographic information in Table 1. SCD participants were recruited within 3 months of the study opening. This component took part between February 2017 and February 2018.

### Mindful colouring-in (pilot attentional condition)

This treatment involved providing basic verbal and written instructions about mindfulness, offering a selection of ‘art therapy’ colouring-in books (frequently available to buy in bookshops in the UK), and introducing guided mindfulness and relaxation techniques. Sessions were conducted on a one-to-one basis in a private room in the secure care unit.

To assess similarities and differences between mindful colouring-in and interpersonal art psychotherapy, audio recordings of mindful colouring-in sessions were rated against the interpersonal art psychotherapy checklist. In comparison to interpersonal art psychotherapy, participants in mindful colouring-in sessions spent more time (proportionately) on art activities, sessions were ‘mindfulness-oriented’ with therapists directing participants accordingly (i.e. awareness of breathing), therapist-client verbal interactions occurred primarily before and after the colouring and/or relaxation activity and time spent in conversation with the therapist was significantly shorter in duration than in interpersonal art psychotherapy.

### Attentional condition acceptability assessment

The acceptability of mindful colouring-in was assessed using multiple SCD studies (*n* = 4) to inform RCT design where an attentional condition [27, 29] might be included as a third arm. Participants (*n* = 4) completed between 10 and 14 individual mindful colouring-in sessions and were then invited to take part in one-to-one semi-structured audio-recorded interviews. A thematic analysis [34] was carried out on interview transcriptions and matched against a conceptual treatment acceptability framework [32]. This mapping exercise identified that mindful colouring-in matched against six out of seven of the treatment acceptability criteria, including (a) affective attitude, with participants giving generally positive feedback about the intervention; (b) burden, an acceptable amount of effort was required to participate; (c) ethicality, the intervention was seen as a fitting activity with participants reporting feelings of control over session time and their level of participation in the sessions; (d) intervention coherence and (e) perceived effectiveness, participants reported understanding the intervention and gave personal examples of how it worked for them, i.e. ‘distraction’, ‘relaxation’ and ‘reducing angry feelings/agitation’; (f) self-efficacy, with participants reporting having confidence in performing tasks inside and outside of the sessions; and no themes could be matched to (g) opportunity costs, for example, comments were not made by participants relating to the extent to which they felt that any benefits or values must be given up in order to engage with the intervention [32].

### Trial capacity (survey)

The aim of the survey was to identify the capacity for qualified art psychotherapists working in secure care settings in the UK to take part in a future trial. The survey was disseminated via the British Association of Art Therapists (BAAT) (2426 members), including the Art Therapy and Learning Disability Special-Interest Group (ATLDSIG) (88 members), and the Forensic Arts Therapies Advisory Group (FATAG) (468 members inclusive of art, dance movement, drama and music therapists) with a closing date in January 2018.

From the 27 survey respondents, 26 met the criteria of being UK Health and Care Professions Council (HCPC) registered art psychotherapists; 62% (16/26) worked in NHS Secure Care, 27% (7/26) Prison Service, 11% (3/26) Private/other secure care providers. There was no duplication of respondents from the same institution. A total of 88% (23/26) of therapist were willing to take part in a future art psychotherapy research study with 84% (22/26) willing to follow a structured treatment manual (i.e. interpersonal art psychotherapy) if trained to do so. Art psychotherapists who indicated that they would be willing to participate in a future trial using a manualised treatment (*n* = 22) routinely provided between 3 and 20 individual art psychotherapy sessions per week (Mdn = 7.5, IQR = 7) often in addition to their work providing group based treatment. Willing respondents estimated that up to 1051 (Min/Max 6–180, Mdn = 22, IQR = 70) patients/prisoners within the secure care settings they worked in would meet minimum eligibility requirements for accessing art psychotherapy as a treatment. From this, total 341 patients/prisoners were estimated as having either low intellectual functioning or an intellectual disability.

## Discussion

The purpose of this study was to assess the feasibility of conducting an RCT of interpersonal art psychotherapy with adults who have an intellectual disability in secure care. Study procedures enabling recruitment and retention at follow-up appear to be viable within secure care settings. The target of recruiting 20 participants to the trial from multiple sites was achieved within 8 months of the study opening. There were no substantive amendments to the published protocol. In a future trial, adjustment is required to our systems for live data-collection monitoring which could be rectified though greater resources being available to the research team.

We believe that the present protocol could be scaled-up to a fully-powered randomised controlled trial.

Change estimates themselves do not form part of the decision to move to a definitive trial but are merely presented for information. Given the imprecisions that results from small feasibility samples [62], we have taken a cautious approach. Our calculation of sample size is based upon minimum important clinical difference, this being a clinical judgement and not taken as the difference observed in any feasibility work.

Importantly, ethical considerations did not emerge during the study with patients detained under the Mental Health Act [35] feeling free to decline their participation in the study with no indication of any confusion or coercion. Data collection for the MOAS achieved a high rate of completion, feasible to use as a primary outcome measure. In a future trial, the MOAS could be used as a screening assessment during enrolment with a lower threshold score being set for eligibility in order to exclude patients with no observed aggressive behaviours. Our observations of participants completing the ICECAP-A indicated that further work is needed to consider the support people with intellectual disabilities might need to complete it. With suitable adaptations to this measure, it is possible that future within-trial cost-effectiveness analysis could be conducted [63]; for example, focusing on the incremental costs of providing art psychotherapy relative to usual care.

Treatment fidelity checks showed study therapists as achieving 82% adherence to the interpersonal art psychotherapy manual and a preliminary assessment of therapeutic alliance indicated measurable gains within early sessions.

From both arms, including delayed treatment, participants who started interpersonal art psychotherapy achieved a high completion rate of 94% (17/18). We recruited a number of participants in the study (30%) who had a dual diagnosis of intellectual disability and schizophrenia or paranoid schizophrenia, schizophrenia being identified a factor associated with higher rates of inpatient violence [5]. Participants with an additional diagnosis such as autistic spectrum disorder completed treatment and engaged in the structured approach to manualised session delivery.

Whilst this study was not statistically powered to detect between group differences, we believe that a ‘signal’ for the potential of interpersonal art psychotherapy was indicated in our post-test comparison with the delayed treatment control. This is shown in a between group effect-size of .62 for total MOAS scores and .93 in the verbal aggression sub-scale, with improvements in reduced verbal aggression potentially indicating the successful targeting of ‘interpersonal’ antecedents of physical aggression. Participants distress related to their mental health symptoms, as measured by the BSI-PSDI showed a between group effect-size of .79. We believe that these signals from the data indicate potential merit in interpersonal art psychotherapy for the reduction of aggressive behaviour and improvements in patient’s self-reported distress [64].

A national survey conducted across secure care provision in the UK indicated that there is both willingness and capacity amongst suitably qualified and registered art psychotherapists to support a future trial.

## Conclusions

This protocol, with modifications to screening procedures and data collection procedures, can be extended to a fully powered trial. Recruitment and retention rates were shown to be acceptable. Interpersonal art psychotherapy was found to bring about improvements in observed aggression and distress related to mental health symptoms in adults with intellectual disability in secure care. Positive engagement in interpersonal art psychotherapy from patients with an additional diagnosis of schizophrenia are promising, particularly as this is a factor associated with higher levels of aggression in inpatient care [5]. Observations of reduced verbal aggression in the treatment group may also provide some indication that interpersonal factors associated with aggression were suitably targeted in the intervention. Based upon the present design, a fully powered study is recommended to assess the effectiveness of interpersonal art psychotherapy.

## Data Availability

Due to this being a feasibility study the primary outcomes, feasibility objectives are reported. Outcome measure datasets for this study are small and can be made available via requests to the corresponding author. Datasets will be retained at Newcastle University.

## Abbreviations

AHPs: Allied health professionals
BSI: The Brief Symptom Inventory
CPA: Care Programme Approach
GAS: Glasgow Anxiety Scale for people with ID
HCPC: Health and Care Professions Council
I-AP: Interpersonal Art Psychotherapy
ICECAP-A: ICEpop CAPability Quality of Life measure for Adults
MDT: Multi-disciplinary team
MOAS: The Modified Overt Aggression Scale
NAS: Novaco Anger Scale
NHS: National Health Service
NICE: National Institute for Health and Care Excellence
NIHR: National Institute for Health Research
PBS: Positive behaviour support
RCT: Randomised controlled trial
REC: Research Ethics Committee
SD: Standard deviation
SCD: Single-case design
WAI: Working Alliance Inventory
