# Correction to: A randomised controlled feasibility study of interpersonal art psychotherapy for the treatment of aggression in people with intellectual disabilities in secure care

**DOI:** 10.1186/s40814-020-00743-6

**Published:** 2020-12-18

**Authors:** Simon S. Hackett, Ania Zubala, Katie Aafjes-van Doorn, Thomas Chadwick, Toni Leigh Harrison, Jane Bourne, Mark Freeston, Andrew Jahoda, John L. Taylor, Cono Ariti, Rachel McNamara, Lindsay Pennington, Elaine McColl, Eileen Kaner

**Affiliations:** 1 Faculty of Medical Sciences, Newcastle University, Newcastle upon Tyne, UK; 2 Cumbria, Northumberland, Tyne & Wear NHS Foundation Trust, Newcastle upon Tyne, UK; 3 University of the Highlands and Islands, Inverness, UK; 4 Yeshiva University, New York, USA; 5 Glasgow University, Glasgow, UK; 6 Northumbria University, Newcastle upon Tyne, UK; 7 Centre for Trials Research, Cardiff University, Cardiff, UK

**Correction to: Pilot Feasibility Stud 6, 180 (2020)**

**https://doi.org/10.1186/s40814-020-00703-0**

Following publication of the original article [1], the authors reported an error in author 3’s last name. The correct last name should be “Aafjes-van Doorn” instead of Aafjes-van Doorm.

The original article has been corrected.
